# Up-regulation of peripheral and central CGRP expression combined with subchondral bone remodeling in rat MIA-induced TMJOA model

**DOI:** 10.22514/jofph.2025.078

**Published:** 2025-12-12

**Authors:** Liqin Xu, Henghua Jiang, Qijun Xu, Wei Fang

**Affiliations:** ^1^State Key Laboratory Breeding Base of Basic Science of Stomatology (Hubei-MOST) & Key Laboratory of Oral Biomedicine Ministry of Education (KLOBM), School and Hospital of Stomatology, Wuhan University, 430079 Wuhan, Hubei, China; ^2^Department of Plastic Surgery, The Third Hospital of Wuhan, 430060 Wuhan, Hubei, China; ^3^Department of Oral and Maxillofacial Surgery, School and Hospital of Stomatology, Wuhan University, 430079 Wuhan, Hubei, China

**Keywords:** Osteoarthritis, Subchondral bone, Pain, Calcitonin gene related peptide, Monosodium iodoacetate, Temporomandibular joint

## Abstract

**Background**: Temporomandibular joint osteoarthritis (TMJOA) is a pathological condition marked by subchondral bone remodeling. Osteoarthritis can lead to TMJ pain, nevertheless, the relationship between nociceptive mechanisms and subchondral bone in TMJOA still unclear. **Methods**: In the present investigation, a rat TMJOA model was established via intra-articular administration of monosodium iodoacetate (MIA). Following the induction of MIA-triggered TMJOA, tissue samples were collected from the TMJ condyle, trigeminal system components including ganglion (TG) and nucleus caudalis (TNC), and hippocampal formation. Micro-computed tomography (Micro-CT) was employed to evaluate subchondral bone degeneration in the TMJ, while tartrate-resistant acid phosphatase (TRAP) staining was conducted to measure the activity of osteoclasts in the subchondral bone. Furthermore, immunofluorescence (IF) staining was performed to detect the expression of calcitonin gene related peptide (CGRP) in the TMJ subchondral bone. Afterwards, immunohistochemistry (IHC) was used to detect the expression of CGRP in the TG, TNC and hippocampus tissues. The experimental results were expressed as mean ± Standard Error of the Mean (SEM) values and two-way Analysis of Variance (ANOVA) with Student-Newman-Keuls *post hoc* testing was employed for statistical comparisons, adopting a significance threshold of *p* < 0.05. **Results**: Compared with the control group, Micro-CT results revealed progressive condylar degeneration over time. Consistently, MIA-induced TMJOA rats demonstrated a pronounced accumulation of TRAP-positive osteoclasts in the subchondral bone. The expression of CGRP in the TMJ subchondral bone, TG, TNC and hippocampus tissues was also obviously increased in MIA-induced TMJOA rats. **Conclusions**: MIA-induced rat TMJOA pain could be attributed to the augmented exprssion of CGRP in the TMJ subchondral bone, TG, TNC and hippocampus tissues. An elevated level of CGRP stimulated nociception which was implicated in the development of peripheral and central sensitization in TMJOA pain.

## 1. Introduction

Temporomandibular joint osteoarthritis (TMJOA) pain is the most prevalent complaint of patients to seek treatment, which is caused by local tissue injury or infection [1]. TMJOA pathology is characterized by chronic inflammation of synovium, progressive cartilage degradation and subchondral bone remodeling [2]. However, the pathophysiology of TMJOA pain is still unclear, especially in subchondral bone.

Clinical research indicates a close association between TMJOA pain and subchondral bone changes. During the TMJOA pain progressive phase, a decrease in subchondral bone volume and density may be found, which reflects active subchondral bone resorption. If the condyle contour and subchondral bone resorption are relatively stable, TMJOA pain is significantly relieved [3].

Previous studies show that subchondral bone remodeling plays an important role in nociception [4] and capsaicin-sensitive sensory neurons contribute to bone lesions in MIA-induced OA model [5]. The number and function of osteoclasts are closely related to the subchondral bone lesions. The differentiation and maturation of osteoclasts can promote the dissolution of inorganic minerals and organic collagen, leading to subchondral bone lesions [6]. The inhibition of subchondral bone lesions should be a key target in the management of OA joint pain [7]. While the relationship between nociceptive mechanism and subchondral bone in TMJOA is not clear.

It has been found that pathological changes in the peripheral nociceptive system around the OA joint are related to pain. Calcitonin gene related peptide (CGRP) is proposed to contribute to pain transmission [8] and plays an important role in OA [9]. As an essential neurotransmitter and modulator, CGRP is extensively distributed in both the peripheral and central nervous, playing a crucial role in the transmission and regulation pain signals [10].

CGRP can promote osteoclast generation, inhibit osteoblast activity, accelerate subchondral bone resorption and degenerative remodeling through autocrine or paracrine modes [11]. Current research highlights the potential role of CGRP in nociceptive pathways, although direct evidence of its presence in the subchondral bone of TMJOA has not been conclusively demonstrated.

The peripheral trigeminal ganglion (TG) served as the primary nociceptive relay station for oral and maxillofacial sensory input and transmitted nociceptive signals to the brainstem’s trigeminal nucleus caudalis (TNC) and ultimately to the hippocampus [12, 13]. CGRP released from primary sensory neurons of the TG, is an important neurotransmitter involved in peripheral and central sensitization [14]. The TNC and hippocampus, as key components of the central nervous system, are vital for pain signal transmission and memory [15, 16]. However, the spatiotemporal expression patterns of CGRP within TG, TNC and hippocampal regions during TMJOA progression require systematic investigation.

To replicate human TMJOA histopathological features and investigate specific nociceptive mechanisms, researchers have developed a monosodium iodoacetate (MIA)-induced animal model [17]. Intraarticular MIA can result in a progressive loss of bone mineral density, resorption of calcified cartilage and subchondral bone, as well as pain [18]. This model can be used to explore the development of neuropathic pain, ongoing pain, and central sensitization [19]. It is important to understand how histologic changes are correlated with the onset of pain. However, to our knowledge, no studies have yet explored the relationship between the nociceptive mechanism and subchondral bone.

## 2. Materials and methods

The animal experiments conducted in this study received formal ethical approval (No. S0792203059) from the Institutional Animal Care and Use Committee at Wuhan University School of Stomatology.

### 2.1 Induction of TMJOA

Forty-eight male Sprague-Dawley rats (8 weeks old), sourced from the Hubei Provincial Experimental Animal Center, were randomly assigned to control and experimental groups, with 8 rats in each 2-week, 4-week and 6-week subgroup. Injections were performed percutaneously targeting the superior articular compartment of rat TMJ. The control groups received 50 μL saline, and the experimental groups received 1 mg MIA dissolved in 50 μL saline. Pain behavior was conducted through the head withdrawal threshold (HWT) assessed by utilizing an electronic von Frey filament.

### 2.2 Tissue harvest and processing

At 2, 4, and, 6 weeks post-injection, rats from both groups were euthanized via isoflurane overdose (n = 8 per group). At each time point, one side of the TMJ condyle tissues were dissected for Micro-CT scanning, and the contralateral TMJs for histopathological and immunofluorescence evaluations, 4 μm thickness tissue sections were utilized. Meanwhile, the TG, TNC and hippocampus tissues underwent fixation and paraffin embedding, and serial 4 μm sections for immunohistochemical analysis.

### 2.3 Micro-CT analysis

The trabecular microstructure was evaluated using a Micro-CT system (μCT50, Scanco Medical, Bassersdorf, Switzerland) operating at 70 kV and 114 μA. Three-dimensional reconstructions were performed in medium resolution mode (15 μm slice thickness) to systematically quantify key morphometric parameters including trabecular number (Tb.N), trabecular thickness (Tb.Th) and trabecular separation (Tb.Sp).

### 2.4 Histological and immunofluorescence analysis

After dewaxing, rehydration and washing, the TMJ condyle sections were treated with tartrate-resistant acid phosphatase (TRAP, Sigma 387-A, St. Louis, MO, USA) according to the manufacturer’s protocol. TRAP staining was conducted to assess subchondral bone osteoclast activity. For histological analysis, TRAP-positive cells were quantified in five randomly chosen microscopic fields using an Olympus microscope. Two independent observers performed the counts, with the mean value from these observations calculated as the final count per section.

According to the previous experimental methods, immunofluorescence staining for CGRP was performed. The TMJ condyle sections underwent antigen retrieval using pepsin (DIG-3009; Maixin, Fuzhou, Fujian, China) and 3% H_2_O_2_ (hydrogen peroxide) at 37 °C for 30 minutes, followed by serum treatment (ZLI-9022; Zhongshan Biotechnology, Beijing, China) for 60 minutes. The sections were then incubated overnight at 4 °C with a mouse anti-CGRP antibody (dilution 1:100; ab81887, Abcam, Cambridge, MA, USA). Then the sections were incubated with Cyanine3 (Cy3) goat anti-mouse Immunoglobulin G (IgG) antibody (dilution 1:100, AS1111, Aspen, CO, USA) for 1 h at 37 °C and the nuclei were stained with 4′-6-diamidino-2-phenyl-indole (DAPI, E607303, Biotechnology, Shanghai, China) for 10 min. Finally, all the samples were washed three times with Phosphate-Buffered Saline (PBS), mounted, and examined under fluorescence microscope by two inspectors.

### 2.5 Immunohistochemistry

CGRP immunohistochemical staining was conducted following the previous study methods. After dewaxing, rehydration and washing, the TG, TNC and hippocampus tissue sections underwent sequential antigen retrieval treatment with 3% H_2_O_2_ (30 minutes) and pepsin digestion (30 minutes). Subsequently, tissue sections were immunostained using goat-derived anti-CGRP antibodies (1:2000 dilution, ab36001, Abcam, Cambridge, MA, USA) through overnight cold incubation at 4 °C. After washing with PBS, tissue sections were incubated with a commercial immunohistochemical detection kit (SP-9001, Zhongshan Biotechnology, Beijing, China) at 37 °C for 30 minutes. The bound immunocomplex was visualized under a microscope by immersing the sections in 3,3′-diaminobenzidine (DAB-0031, Maixin, Fuzhou, Fujian, China) for 2 min. Finally, the sections were counterstained with hematoxylin (0000259613; Sigma, St. Louis, MO, USA) and mounted with mounting medium. For quantitative analysis, two independent examiners randomly selected five visual fields at 400× magnification under standardized microscopic observation.

### 2.6 Data analysis

The experimental results were expressed as mean ± SEM values processed through GraphPad Prism 6.0 software (GraphPad Software, Inc., San Diego, CA, USA). Two-way ANOVA with Student-Newman-Keuls *post hoc* testing was employed for statistical comparisons, adopting a significance threshold of *p* < 0.05.

## 3. Results

### 3.1 The TMJ subchondral bone remolding

No bony changes among the control groups were observed. Compared with the control groups (Fig. 1A-a), bone lesions were initially observed and worsened at 2 weeks (Fig. 1A-b) and gradually recovered at 4 and 6 weeks (Fig. 1A-c,d) in the experimental groups. Osteophytes were observed at 6 weeks (Fig. 1A-d). Micro-CT analysis revealed that Tb.N and Tb.Th decreased, whereas Tb.Sp increased in the experimental groups. The most evident changes were observed at 2 weeks group (Fig. 1B–D).

**Fig. 1.** Bony changes in TMJ condyle. (A) In the control group (a), the subchondral bone was regularly aligned. Subchondral bone loss (arrows) were observed in the experimental groups ((b) 2 weeks MIA injection group, (c) 4 weeks MIA injection group). Osteophytes (arrows) were observed at 6 weeks MIA injection group (d). Lesion regions were larger at 2 weeks (b) than those at other time points (c, d). Scale bar = 1.0 mm in (A). Micro-CT analysis revealed that Tb.N (B) and Tb.Th (C) decreased, whereas Tb.Sp (D) increased at 2 weeks groups (*p < 0.05, **p < 0.01, ***p < 0.001). Tb.N: trabecular number; Tb.Th: trabecular thickness; Tb.Sp: trabecular separation; CT: computed tomography.

### 3.2 Osteoclast in the TMJ subchondral bone

In the control groups, few TRAP-positive cells were observed in the subchondral bone. No statistical significance among the control groups was observed. While TRAP-positive cells in the subchondral bone were significantly upregulated after MIA injection in the experimental groups, including 2-week, 4-week, and 6-week (Fig. 2A). In the experimental groups, TRAP-positive cells demonstrated a biphasic pattern, peaking at 2 weeks followed by gradual regression through 4- and 6-weeks groups (Fig. 2B).

**Fig. 2.** TRAP-positive cells in the TMJ subchondral bone. (A) In the control groups (a), TRAP staining showed that few osteoclast cells were observed in the subchondral bone. While, TRAP-positive cells in the subchondral bone were upregulated after MIA injection in the experimental groups, including 2, 4 and 6 weeks ((b) 2 weeks MIA injection group, (c) 4 weeks MIA injection group, (d) 6 weeks MIA injection group; scale bar = 50 μm in (A)). (B) In the experimental groups, TRAP-positive cells demonstrated a biphasic pattern, peaking at 2 weeks followed by gradual regression through 4- and 6-weeks groups (*p < 0.05, ***p < 0.001). TRAP: tartrate-resistant acid phosphatase.

### 3.3 CGRP expression in the TMJ subchondral bone

The expression of CGRP in the subchondral bone was studied by immunofluorescence. CGRP was localized in the cytoplasm of osteoclasts. In the control groups, few CGRP-positive osteoclasts were observed in the subchondral bone. While CGRP-positive osteoclasts were obviously observed after MIA injection in the experimental groups, including 2-week, 4-week, and 6-week (Fig. 3A). As the number of osteoclasts was increased, the expression of CGRP in the subchondral bone was also upregulated accordingly. In the experimental groups, CGRP-positive osteoclasts demonstrated a biphasic pattern, peaking at 2 weeks followed by gradual regression through 4- and 6-weeks groups (Fig. 3B).

**Fig. 3.** CGRP-positive osteoclasts in the TMJ subchondral bone. (A) CGRP was localized in the cytoplasm of osteoclasts. In the control groups, immunofluorescence staining showed that few CGRP-positive osteoclasts were observed in the subchondral bone. While CGRP-positive osteoclasts were obviously observed after MIA injection in the experimental groups, including 2, 4 and 6 weeks. Scale bar = 25 μm in (A). (B) In the experimental groups, CGRP-positive osteoclasts demonstrated a biphasic pattern, peaking at 2 weeks followed by gradual regression through 4- and 6-weeks groups (*p < 0.05, ***p < 0.001). CGRP: calcitonin gene related peptide; DAPI: 4′-6-diamidino-2-phenyl-indole.

### 3.4 CGRP expression in the TG

In the control groups, few CGRP-positive cells were positively stained with CGRP in the TG. While CGRP-positive cells were obviously observed after MIA injection in the experimental groups, including 2-week, 4-week, and 6-week (Fig. 4A). In the experimental groups, CGRP-positive cells in the TG demonstrated a biphasic pattern, peaking at 2 weeks followed by gradual regression through 4- and 6-weeks groups (Fig. 4B).

**Fig. 4.** CGRP expression in the TG. (A) In the control groups (a), immunohistochemical staining showed that few CGRP-positive cells were observed in the TG. While CGRP-positive cells were obviously observed after MIA injection in the experimental groups, including 2, 4 and 6 weeks ((b) 2 weeks MIA injection group, (c) 4 weeks MIA injection group, (d) 6 weeks MIA injection group; scale bar = 50 μm in (A)). (B) In the experimental groups, CGRP-positive cells in the TG demonstrated a biphasic pattern, peaking at 2 weeks followed by gradual regression through 4- and 6-weeks groups (***p < 0.001). TG: trigeminal ganglion; CGRP: calcitonin gene related peptide.

### 3.5 CGRP expression in the TNC and hippocampus

No statistical significance among the control groups was observed. Few CGRP-immunoreactive fibers were observed in the TNC and few CGRP-positive cells were observed in the hippocampus. CGRP expression in the TNC and hippocampus was obviously observed in MIA-induced TMJOA rats compared with that in the control groups (Fig. 5A). In the experimental groups, CGRP-positive fibers in the TNC and cells in the hippocampus demonstrated a biphasic pattern, peaking at 2 weeks followed by gradual regression through 4- and 6-weeks groups (Fig. 5B).

**Fig. 5.** CGRP expression in the TNC and hippocampus. (A) In the control groups (a, e), immunohistochemical staining showed that few CGRP-immunoreactive fibers were observed in the TNC (a), and few CGRP-positive cells were observed in the hippocampus (e). While, CGRP expression in the TNC (b–d) and hippocampus (f–h) was significantly increased after MIA injection in the experimental groups, including 2, 4 and 6 weeks (b/f: 2 weeks MIA injection group, c/g: 4 weeks MIA injection group, d/h: 6 weeks MIA injection group; scale bar = 50 μm in (A)). (B) In the experimental groups, CGRP-immunoreactive fibers in the TNC (Fig. 5B-a) and CGRP-positive cells in the hippocampus (Fig. 5B-b) demonstrated a biphasic pattern, peaking at 2 weeks followed by gradual regression through 4- and 6-weeks groups (***p < 0.001). TNC: trigeminal nucleus caudalis; CGRP: calcitonin gene related peptide.

## 4. Discussion

The intra-articular MIA administration method demonstrated effective reproducibility for establishing OA models. A previous study in my research group showed that TMJ cartilage degeneration and chronic pain behavior were observed at 2 weeks post-MIA injection [4]. In this study, an MIA dose of 1 mg was used to build TMJOA animal model. Obvious subchondral bone lesions were found with the increasing number of osteoclasts at 2 weeks and gradually recovered at 4 and 6 weeks. During OA, increased osteoclast and osteoblast activity were found in the subchondral bone, which could potentially be a source of pain [20]. In this study, following the administration of MIA, an increased TRAP-positive cells were observed in the subchondral bone, with this elevation peaked at 2 weeks. Intraarticular MIA injection resulted in significant pain behavior and increased numbers of subchondral osteoclast [5]. In general, osteoclast created a closed acidic microenvironment and then resorbed bone minerals [21]. In acidic microenvironments, sensory neurons exhibited heightened sensitivity through activation of acid-sensing ion channels (ASICs), which function as specialized proton detectors in peripheral nociceptive pathways [22, 23]. Inhibition of osteoclast function was found to alleviate pain. Pain might be improved by bisphosphonate through inhibiting osteoclast function [24]. Restricting osteoclast activity could potentially alter subchondral bone abnormal remodeling as well as decrease pain behavior in the early phase of OA [6]. Osteoclast-specific Netrin-1 deletion may prevent sensory nerve fibers from entering porous endplates, thereby alleviating spinal hypersensitivity [25].

CGRP, an important neuropeptide, is extensively distributed in peripheral and central neurons, exhibiting potent analgesic and vasodilatory effects [26]. A higher expression of CGRP was observed in the hip OA model [27]. In this study, CGRP expression in the TMJ subchondral bone osteoclasts was upregulated after MIA injection. CGRP might regulate load-induced skeletal repair responses through neural signaling pathways [28], and compression significantly increased CGRP concentrations in bone [29]. CGRP mediated subchondral bone remodeling by modulating the OPG/RANKL (osteoprotegerin/receptor activator of Nuclear Factor kappa B cells (NF-κB) ligand) ratio, a critical determinant in osteoclast differentiation and bone resorption processes [30, 31]. As a key regulator in bone homeostasis, CGRP enhanced bone formation by stimulating stromal cell differentiation into osteoblasts, while concurrently suppressing bone resorption through inhibiting RANKL expression [30, 32]. In this study, the expression of CGRP in subchondral bone osteoclasts reached a peak at 2 weeks. The upregulation of CGRP indicated the strong amplification and transmission of pain signals in TMJOA.

In the MIA-induced OA model, pain behavior might initially arise from an inflammatory pain, which subsequently evolves into neuropathic pain through progressive neuronal damage [33]. Inflammation of the peripheral target tissue cause CGRP expression, which could promote peripheral sensitization throughout the ganglion [34]. The upregulation of CGRP in synovium might participate in the inflammatory process of arthritis [35].

Various studies have employed Complete Freund’s Adjuvant (CFA) to establish a TMJ inflammatory model, thereby investigating the changes in the TG and the central nervous system (CNS) [36]. CFA-induced TMJ inflammation tissues exhibited marked elevation levels of interleukin-1β (IL-1β) and CGRP [37], and chronic low-grade inflammation might play a pivotal role in mediating both nociceptive signaling and degenerative alterations in TMJ [38].

It had been shown that CGRP expression was increased in DRG neuron. DRG and TG sensory neurons exhibited a distinctive bidirectional neuropeptide signaling in both peripheral and central axonal terminals [33], which affecting blood flow, inflammatory responses, and nociceptive signals [37]. In this study, CGRP expression in the TG was upregulated following MIA injection.

Once synthesized in the TG neurons, CGRP was stored in dense core vesicles within the peripheral and central nerve terminals [39], which enhance the propagation of inflammatory signals throughout the ganglion and promote peripheral sensitization [40].

As nociceptive stimulation signals in the TMJ subchondral bone were transferred from TG to TNC and finally reached the hippocampus which is involved in the regulation of emotion and memory [32, 33]. Therefore, it becomes imperative to investigate the neuromodulatory effects of CGRP distributed in trigeminal ganglion (TG), trigeminal nucleus caudalis (TNC) and hippocampus on TMJOA. In this study, CGRP levels in the TG, TNC and hippocampus increased and reached a peak at 2 weeks. Bone lesions and increased numbers of subchondral osteoclast were also initially observed and worsened at 2 weeks and gradually recovered at 4 and 6 weeks. This finding was in line with the characteristics of CGRP distributed in trigeminal ganglion (TG), trigeminal nucleus caudalis (TNC) and hippocampus.

The high expression of CGRP observed in the TG, TNC and hippocampus was coincided with chronic pain behavior, demonstrating that nociceptive stimulation concurrent activation in both the peripheral and central nervous systems. This study provides valuable insights into the mechanism of pain caused by TMJOA. However, the current research has limitations in terms of cell experiments and fails to deeply explore the underlying molecular mechanisms. Future research should explore the molecular mechanisms more thoroughly.

## 5. Conclusions

Collectively, an elevated level of CGRP stimulates nociception which was implicated in the development of TMJOA pain. Rat TMJOA pain could be attributed to the augmented expression of CGRP in the TMJ subchondral bone, TG, TNC and hippocampus tissues.
